# Simultaneous Detection of Classical and African Swine Fever Viruses by Duplex Taqman Real-Time PCR Assay in Pigs Infected with Both Diseases

**DOI:** 10.3390/pathogens14050473

**Published:** 2025-05-13

**Authors:** Liani Coronado, Adriana Muñoz-Aguilera, Miaomiao Wang, Iván Muñoz, Cristina Riquelme, Saray Heredia, Katarzyna Stępniewska, Carmina Gallardo, Llilianne Ganges

**Affiliations:** 1 WOAH Reference Laboratory for Classical Swine Fever, IRTA-CReSA, 08193 Barcelona, Spain; liani.coronado@irta.cat (L.C.); adriana.munoz@irta.cat (A.M.-A.); mioa@tsinghua.edu.cn (M.W.); ivan.munoz@irta.cat (I.M.); cristina.riquelme@irta.cat (C.R.); saray.heredia@irta.cat (S.H.); 2 Unitat Mixta d’Investigació IRTA-UAB en Sanitat Animal, Centre de Recerca en Sanitat Animal (CReSA), Bellaterra, 08193 Barcelona, Spain; 3 IRTA, Programa de Sanitat Animal, Centre de Recerca en Sanitat Animal (CReSA), Bellaterra, 08193 Barcelona, Spain; 4 Instituto Colombiano Agropecuario (ICA), Bogotá 110911, Colombia; 5 Department of Swine Diseases, National Veterinary Research Institute, Partyzantów 57 Avenue, 24-100 Pulawy, Poland; katarzyna.stepniewska@piwet.pulawy.pl; 6 European Union Reference Laboratory for African Swine Fever (EURL), Centro de Investigación en Sanidad Animal (CISA), Instituto Nacional de Investigación y Tecnología Agraria y Alimentaria (INIA), Consejo Superior de Investigaciones Científicas (CSIC), Valdeolmos, 28130 Madrid, Spain; gallardo@inia.csic.es

**Keywords:** early diagnosis, differential detection, duplex qPCR, CSFV, ASFV, doubly infected pigs, surveillance

## Abstract

The increasing spread of African swine fever (ASF) in recent years and the presence of classical swine fever (CSF) subclinical forms in endemic countries suggests that the possibility of coinfection with ASF virus (ASFV) and CSF virus (CSFV) in pigs cannot be ruled out in areas where both diseases are prevalent. Thus, rapid and reliable diagnosis through molecular testing is essential for the timely implementation of control measures to prevent the spread of these devastating swine diseases. Here, we have coupled two of the most validated PCR assays for the detection of CSFV and ASFV in a single reaction tube. The combination of the two tests for the detection of two target nucleic acids did not affect the analytical sensitivity, and the duplex RT-qPCR assay was comparable with the standard molecular techniques. The detection limits for CSFV RNA and ASFV DNA were 0.12 TCID_50_/reaction and 0.25 TCID_50_/reaction, respectively. The test showed high repeatability and reproducibility, the coefficient of variation was below 2%, and excellent performance was demonstrated in clinical samples. The duplex assay shows great potential to become a robust diagnostic tool for the rapid and reliable detection and differentiation of CSFV and ASFV in areas where both viruses may be circulating.

## 1. Introduction

African swine fever (ASF) and classical swine fever (CSF) are devasting viral infectious diseases affecting swine [1,2]. Both diseases are notifiable to the World Organization for Animal Health (WOAH) due to the high mortality rates, rapid spread, and economic losses that generate a negative impact on international trade [3,4]. ASF virus (ASFV), the causative agent of ASF, is a large double-stranded DNA virus with a complex molecular structure, being the only member of the Asfarviridae family [5]. Currently, ASF represents a serious worldwide threat in the absence of a globally available commercial vaccine. Since 2007, to date, ASFV have been circulating and spreading continuously in many countries across Europe and Asia [6,7], and in 2021 it spread to the American continent (Dominican Republic and Haiti) [8,9]. Meanwhile, in Europe, ASF prevalence in wild boars plays a relevant role in the risk of ASFV transmission to the domestic population [7,10].

CSF is caused by CSF virus (CSFV), a highly contagious, small, enveloped, and single-stranded RNA virus belonging to the Pestivirus genus in the Flaviviridae family [11]. Currently, CSFV is mainly found in Central and South America, the Caribbean, and in many Asian countries [12]. Some CSF-endemic countries are currently also affected by ASF [13,14,15]. Considering the similarities of clinical symptoms between both diseases, and the possible occurrence of non-specific clinical symptoms [16,17], rapid and reliable diagnosis through molecular testing is essential for the timely implementation of control measures to prevent the spread of these devastating diseases. This highlights the need for a rapid and effective diagnostic test to support the surveillance programs of these diseases [3].

In the present study, two of the most recommended WOAH PCR assays for the molecular detection for ASFV and CSFV, respectively, were coupled in a single reaction tube for the standardization of the duplex RT-qPCR test. The test was also evaluated, using a wide matrices panel that includes samples collected from CSFV and ASFV experimentally infected animals at different time points. In addition, samples from pigs infected with both were also analyzed. The duplex assay, using TaqMan probes, enabled simultaneous, early differential diagnosis with high accuracy and sensitivity. In this way, the diagnosis of both diseases can be sped up while optimizing costs and ASF and CSF molecular surveillance.

## 2. Materials and Methods

### 2.1. Cell and Viruses

The ASFV Badajoz 71 strain (BA71V, genotype I), isolated from the 1971 Spanish ASFV outbreak, and the ASFV Es15/WB-Valga-14 (genotype II) strain were used. These viruses were provided by the European Union Reference Laboratory (EURL) for ASF, INIA-CISA-CSIC, Madrid, Spain. Notably, the ASFV Es15/WB-Valga-14 strain was previously characterized as a moderate virulence strain [18]. The CSFV Catalonia 01 (Cat01) strain (genotype 2.3) also was used. The porcine kidney cell line PK-15 (ATCC-CCL-33) was used for viral production, and the CSFV strains were grown in Eagle’s minimum essential medium (Lonza, Basel, Switzerland) supplemented with 5% of Pestivirus-free fetal bovine serum (FBS), incubated for 72 h at 37 °C at 5% CO_2_, after cell culture inoculation. Determination of viral titers was carried out by end-point dilution, calculated following standard statistical methods [19]. Viral replication was monitored using Peroxidase-linked assay (PLA) [20].

### 2.2. Nucleic Acid Extraction and CSFV and ASFV Molecular Detection

Viral nucleic acid was extracted from all the viral cultures and infected animal samples for analysis by single CSFV RT-qPCR and ASFV qPCR, and the new duplex RT-qPCR assays. In all cases, an initial sample volume of 200 mL was used for extraction with the MagAttract 96 cador Pathogen Kit (Qiagen, Hilden, Germany), following the manufacturer’s instructions. The supernatant of the tissue samples, previously ground in 900 µL of Eagle’s minimum essential medium, supplemented with 2% penicillin (10,000 U/mL) and streptomycin (10,000 U/mL), and centrifuged at 13,000 RPM for 10 min, was used for nucleic acid extraction. ASFV DNA detection was carried out using the previously described qPCR test [21], using the modified protocol that employs the ASF-VP72P1 probe instead of the UPL probe, in accordance with the WOAH guidelines [4]. CSFV RNA was detected using the RT-qPCR assay [22]. In both tests, samples were considered positive when the threshold cycle (Ct) values were equal or less than 40, and negative when fluorescence was undetectable.

For the duplex qPCR, the primers and probes using CSFV and ASFV [21,22] were added in a single reaction tube. In the case of the assay described by [22], the TaqMan probe (CSF-Probe 1) was modified with a Cy5 quencher. The duplex qPCR assay was optimized side by side with both previously described tests [21,22]. The amplification reactions were carried out in a final volume of 20 µL, using the AgPath-ID™ One-Step RT-PCR Reagents (Applied Biosystems, Waltham, MA, USA). Viral nucleic acid samples (2 µL) were added to a 18 µL master mix containing 10 µM of each probe and 20 µM of each primer. The thermoprofile was selected as follows: reverse transcription at 48 °C for 10 min, followed by incubation at 95 °C for 10 min, five cycles at 95 °C for 1 min and 60 °C for 30 s, and then 50 cycles at 95 °C for 10 s and 60 °C for 30 s with fluorescence reading at the end of each cycle. Fluorescence data were collected on the FAM channel for ASFV and on the Cy5 channel in the case of CSFV. After amplification, a Ct value was assigned to each sample. All runs were conducted using an Applied Biosystems 7500 Fast Real-Time PCR System and QuantStudio 5 Real-Time PCR System (Life Technologies, Carlsbad, CA, USA). During the optimization of the protocols, several experimental steps were conducted to set up the reagent concentrations and the thermocycling parameters.

### 2.3. CSF-ASF Duplex RT-qPCR Analytical Sensitivity

The analytical sensitivity of the optimized duplex RT-qPCR assay was determined to use a log-10 dilution series of viral nucleic acid from one strain of CSFV and one strain of ASFV. The strains, Cat01 (CSFV) and Badajoz (ASFV), with a viral title of 10^5,8^ TCID_50_/mL and 10^6,1^ TCID_50_/mL, respectively, were used. Such serial dilutions were used to establish a standard curve for each target by plotting the threshold cycles with log dilution factors using three technical replications. The sensitivity obtained by the new duplex RT-qPCR assay was compared side by side with the WOAH recommended assays for CSFV or ASFV detection in single format.

### 2.4. Analytic Specificity Determination of the Duplex RT-qPCR Assay

To determine the specificity of the established RT-qPCR assay, the nucleic acid from other viral pathogens relevant to swine health, as well as pathogens genomically related to CSFV and ASFV, including Bovine Viral Diarrhea virus, types I and II (BVDV-I and BVDV-II, respectively), Border Disease Virus (BDV), pseudorabies virus (PRV), Porcine reproductive and respiratory syndrome virus (PRRSV), Porcine circovirus type 2 (PCV2), Porcine parvovirus (PPV), Atypical porcine pestivirus (APPV) and, influenza virus, were used as templates.

### 2.5. Validation of the Duplex RT-qPCR Using Samples from Inter-Laboratory Comparison Test (ILCT) Panels

The validation of the duplex assay was performed using sample panels from the CSF EURL, Hanover, Germany, and the ASF EURL CISA-INIA-CSIC, Madrid, Spain. This includes four ILCT sample panels conducted in 2019 and 2020, two ASF reference panels, and another two from CSF (Table 1). Each panel includes positive serum samples with different viral loads, obtained from experimental infections in pigs, as well as a negative commercial pig serum. All samples were evaluated in duplicate and compared with the ILCT results.

### 2.6. Duplex RT-qPCR Validation in Samples from Experimentally Infected Pigs

Samples obtained from animals experimentally infected with either ASFV or CSFV were used for duplex RT-qPCR assay validation. Samples from pigs infected with both viruses were also included. Different types of matrices, including serum, blood, nasal and rectal swabs, mesenteric lymph nodes, tonsils, spleens, and muscles, were used to validate the duplex RT-qPCR. A total of 36 samples were collected from CSFV Cat01-strain-infected pigs at 7, 14, and 21 dpi [23]. The same types of samples, 52 in total, were also collected from ASFV-infected pigs at 3, 7, and 13 dpi, that were infected with the Es15/WB-Valga-14 (genotype II) strain, using 20 hemadsorption units (HA_50_)/mL, through intranasal inoculation. In addition, samples from pigs that were CSFV and ASFV co-infected were also included [23,24]. CSFV persistent infected animals with the Cat01 strain [23] were inoculated at 35 dpi using 20 HA_50_/mL of ASFV Es15/WB-Valga-14 strain. Forty-eight samples were used from these animals at 3 and 7 dpi. The experiments were carried out in biosafety level 3 facilities (BSL3) at IRTA-CReSA, according to existing Spanish and European regulations. The protocol had been approved by the Ethical Committee of the Generalitat de Catalonia, Spain, under the animal experimentation project number 10789. All samples were processed and evaluated by the single CSFV or ASFV molecular tests, as well as by the duplex assay.

### 2.7. Reproducibility of the Duplex RT-qPCR

To assess the intra-assay repeatability and inter-assay reproducibility of the duplex RT-qPCR assay, high, medium and low doses of CSFV RNA and ASFV DNA (10^3^, 10^2^, and 10 TCID_50_ per reaction) were tested in triplicate in one run or in three independent runs on different days. The intra- and inter-assay coefficients of variation (CV) for the Ct values were calculated following the formula CV = (SD [Ct-value]/overall mean [Ct-value]) × 100, in accordance with previously published guidelines (U.S. Environmental Protection Agency, 2004).

## 3. Results

### 3.1. Analytical Sensitivity of the Duplex RT-qPCR Assay

**Figure 1:**
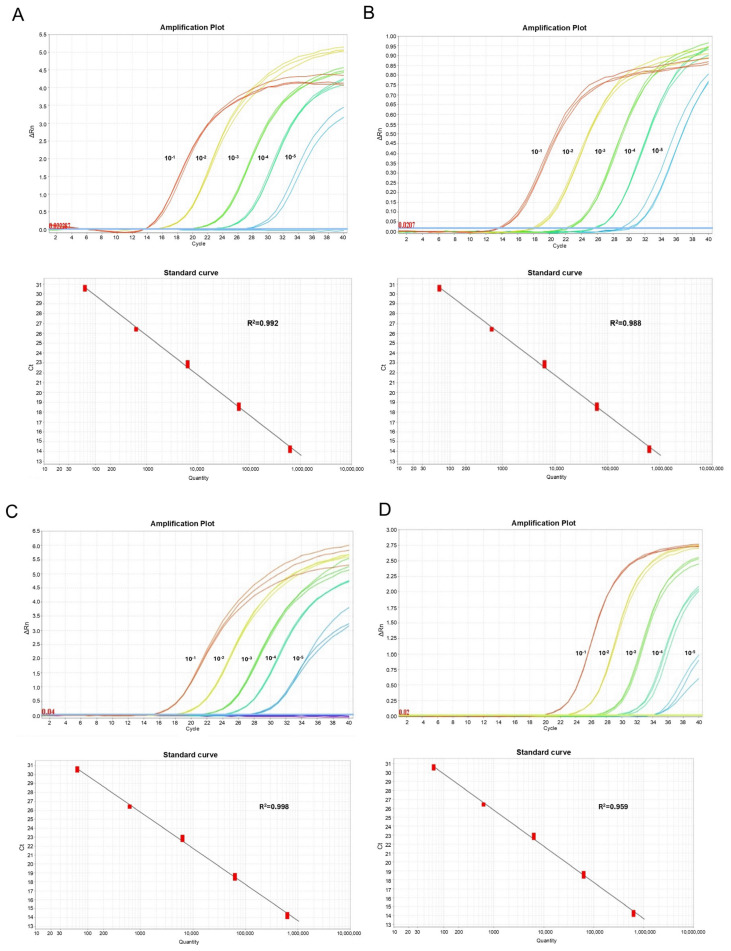
Sensitivity and standard curves of duplex RT-qPCR compared with standard tests using 1:10 serial dilutions of CSFV and ASFV strains. (**A**) CSFV target in singleplex assay, (**B**) CSFV target in duplex assay, (**C**) ASFV target in singleplex assay, and (**D**) ASFV target in duplex assay.

The analytical sensitivity of the new duplex RT-qPCR test was determined using serial tenfold CSFV strain and ASFV strain dilutions. The detection limit of the duplex RT-qPCR assay in cell culture medium was 0.12 TCID_50_/reaction for CSFV using the Cat01 strain, and 0.25 TCID_50_/reaction for ASFV using the BA71V strain. The limits of detection and the amplification efficiencies were not affected by the presence of two primer pairs and two probes in a single-reaction tube. The Ct-value at the detection limit was determined to be 40.0 for the new duplex RT-qPCR system for both viral targets. The sensitivity results of the duplex RT-qPCR method in comparison with the recommended WOAH used assays for CSFV and ASFV detection are shown in Figure 1. The trendline for both viral agents showed a high degree of linearity: R^2^ = 0.992 and R^2^ = 0.998 for CSFV and ASFV, respectively (Figure 1).

### 3.2. Analytical Specificity of the Duplex RT-qPCR Assay

For the specificity analysis, the nucleic acid of different porcine viruses was used as a template for the newly developed duplex RT-qPCR. As a result, only ASFV and CSFV showed amplification curves. The other viruses, including PRV, PRRSV, PCV2, PPV, APPV, influenza virus, BDV, and BVDV, did not show any fluorescent signals or amplification curves.

### 3.3. Validation of the Duplex RT-qPCR Assay

**Figure 2:**
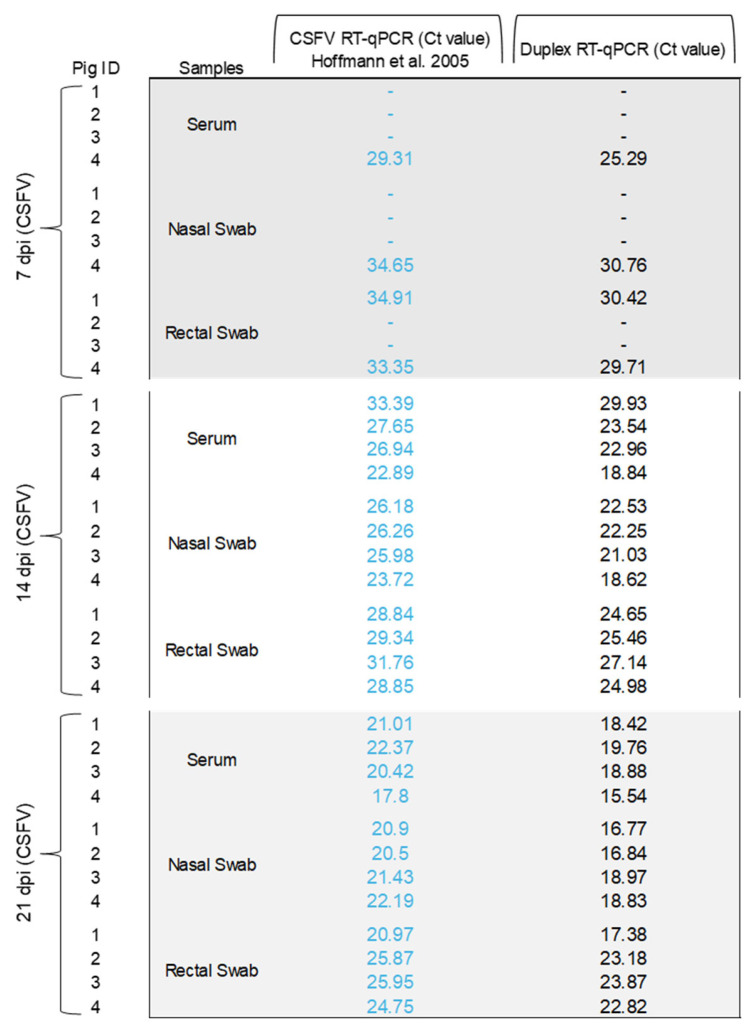
Detection of CSFV in clinical samples from experimental infected pigs using the duplex RT-qPCR assay. The Ct values obtained using CSFV RT-qPCR [22] are shown in blue. The Ct values obtained using CSFV-ASFV duplex RT-qPCR are shown in black.

**Figure 3:**
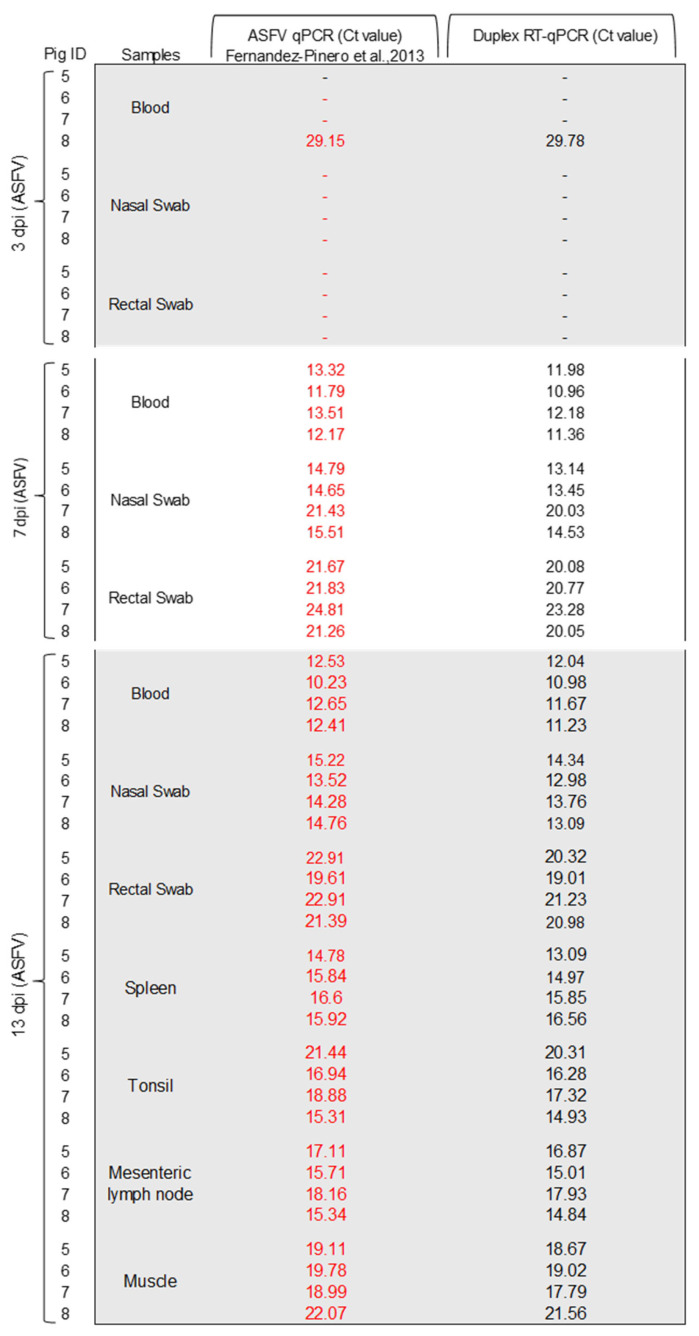
Detection of ASFV in clinical samples from experimentally infected pigs using the duplex RT-qPCR assay. The Ct values obtained using ASFV qPCR [21] are shown in red. The Ct values obtained using CSFV-ASFV duplex RT-qPCR are shown in black.

**Figure 4:**
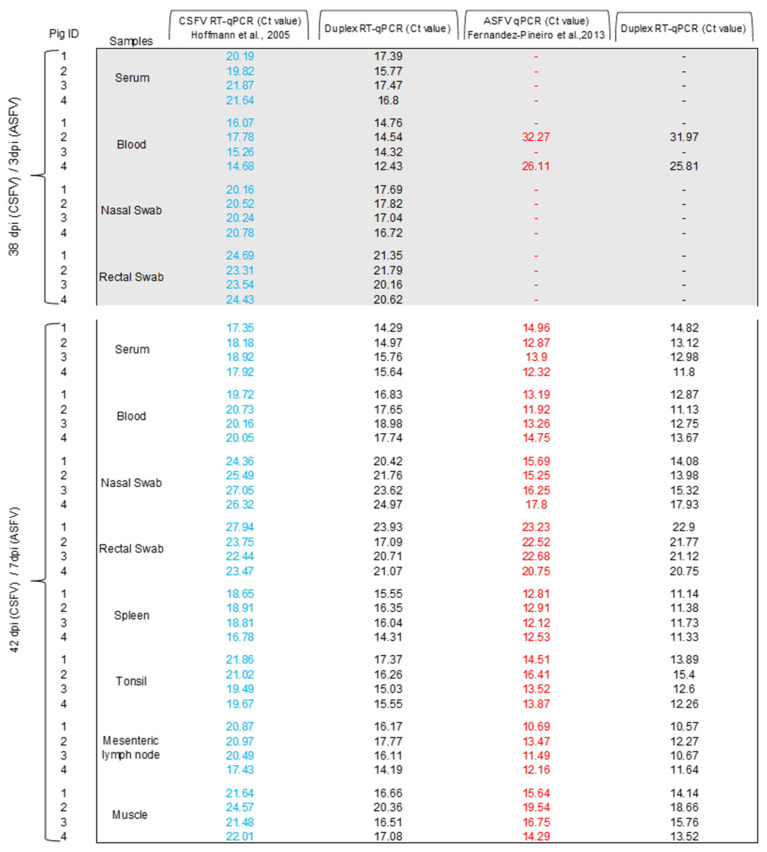
Detection of CSFV and ASFV in clinical samples from experimental co-infected pigs using the duplex RT-qPCR assay. The Ct values obtained using CSFV RT-qPCR [22] are shown in blue. The Ct values obtained using ASFV qPCR [21] are shown in red. The Ct values obtained using CSFV-ASFV duplex RT-qPCR are shown in black.

The results of the new duplex RT-qPCR assay for the simultaneous detection of CSFV and ASFV applied to standard clinical samples were consistent with the expected results (100%) of the ILCT (Table 1). Likewise, this assay was compared side by side with the single CSFV and ASFV tests for both diseases, using clinical samples collected from animals experimentally infected with CSFV or ASFV, and CSFV-ASFV co-infected animals. In the 36 samples evaluated from CSFV infected animals, the results obtained using the developed duplex RT-qPCR test were consistent with the results of the CSFV reference molecular test (Figure 2). Likewise, in the 52 samples tested from ASFV-infected animals, the results obtained using the duplex qPCR test were also consistent with the results of the reference procedure (Figure 3). In samples from CSFV-ASFV co-infected pigs, the 36 evaluated samples result in 100% coincidence using the three assays (Figure 4). Therefore, the duplex assay did not affect the simultaneous detection of the two nucleic acids in the same sample. Also, no false-negative or false-positive results were observed.

### 3.4. Intra- and Inter-Assay Variability

The duplex RT-qPCR assay demonstrated high repeatability, with a CV within runs (intra-assay variability) and between runs (inter-assay variability) ranging from 0.41% to 1.20% and 0.34% to 1.62%, respectively. The CV values were all < 2%, indicating that the method has good repeatability and proficiency.

## 4. Discussion

Among the transboundary animal diseases affecting swine, ASF and CSF show indistinguishable clinical forms with high socio-economic consequences [5,15]. The international scenario characterized by numerous outbreaks of ASF in several European and non-European countries [25,26], and the high number of CSF endemic regions [2], increases the probability of finding both viruses circulating in the same area, which may interfere with diagnosis, and therefore with surveillance programs and the establishment of rapid control measures. Considering this, the control of ASF and CSF relies on the establishment of early detection systems which include accurate diagnostic tools.

PCR assays in multiplex format are an excellent choice of diagnostic method because they can rapidly, precisely, sensitively, and accurately identify multiple pathogenic nucleic acids in a single reaction [27]. However, their development is not a straightforward procedure and is more challenging than the design of singleplex qPCR assays. This strategy often requires extensive primer optimization, and non-specific amplicons can interfere with the amplification of desired targets [28].

In the present study, the two assays for the diagnosis of ASF and CSF, also recommended by WOAH and widely validated in most laboratories worldwide, have been combined in a single reaction tube, using the duplex format. This assay showed high sensitivity for both viruses, like the reference RT-qPCR and qPCR assays, demonstrating the ability to precisely detect both CSFV and ASFV at the same time. Likewise, no cross-reactivity with other porcine pathogens was found. Moreover, the reproducibility of the test showed CVs for CSFV and ASFV below 2%, lower than that found in other similar studies [29,30,31]. This shows the high level of specificity, sensitivity, and repeatability of the duplex assay for both CSFV and ASFV.

Furthermore, to evaluate the clinical application of the method, validation experiments using samples from animals experimentally infected with ASFV and CSFV, including animals infected with both viruses, were performed. The overall concordance rate between the duplex RT-qPCR and the single format tests was 100% in the wide panels of the evaluated matrices, confirming that diagnostic sensitivity is maintained despite coupling both assays in a single reaction tube. Notably, in samples from CSFV-ASFV co-infected pigs, the duplex assay was able to detect both nucleic acids without interference, demonstrating the detection capability and the value of the novel duplex assay for routine diagnosis in infected animals.

## 5. Conclusions

In summary, a duplex RT-qPCR has been successfully developed for the simultaneous detection and differentiation of CSFV and ASFV. The test shows excellent specificity, high sensitivity, and good repeatability. This assay combines the tests recommended by WOAH for these transboundary diseases in a single reaction tube, shortening the response time and the human and economical resources for faster detection and differentiation of CSF and ASF in swine. The duplex RT-qPCR was accredited in our laboratory under the ISO/IEC 17025: 2017.

## Figures and Tables

**Table 1:** Evaluation and comparison of the diagnostic performance of duplex RT-qPCR using ASF and CSF reference sample panels.

Virus	ILCT	Reference Strain/ Genotype	Sample ID	(Ct Value) EURL	(Ct Value) Duplex RT-qPCR
ASFV	ASF EURL (2019)	Arm07/II	Nº 1	27.2	24.53
		Arm07/II	Nº 2	27.7	21.38
		Porcine blood negative	Nº 3	undet.	undet.
		Porcine blood negative	Nº 4	undet.	undet.
		Arm07/II	Nº 5	27.2	24.66
		Arm07/II	Nº 6	23.9	20.29
		Arm07/II	Nº 7	20.09	17.02
		Arm07/II	Nº 8	27.7	23.65
		Arm07/II	Nº 9	23.9	20.82
		Arm07/II	Nº 10	20.09	17.32
	ASF EURL (2020)	Arm07/II	03-01	23.9	21.19
		Arm07/II	03-02	27.5	25.33
		Porcine blood negative	03-03	undet.	undet.
		Arm07/II	03-04	20.7	17.55
		Arm07/II	03-05	23.9	21.06
		Arm07/II	03-06	27.5	24.04
		Porcine blood negative	03-07	undet.	undet.
		Arm07/II	03-08	20.7	17.14
		Arm07/II	03-09	23.9	21.82
		Arm07/II	03-10	27.5	25.49
CSFV	CSF EURL (2019)	CSF1053(21dpi)/2.3	Viro A	33	26.52
		CSF0864(25dpi)/2.3	Viro B	27	26.06
		CSF1053(21dpi)/2.3	Viro C	33	26.83
		CSF1045(17dpi)/2.3	Viro D	21	15.67
		Porcine blood negative	Viro E	undet.	undet.
		CSF0309/3.4	Viro F	31	23.81
		CSF1047/2.1	Viro G	18	14.43
		Porcine blood negative	Viro H	undet.	undet.
	CSF EURL (2020)	Koslov/1.1	Viro A	22	16.13
		CSF1060(14dpi)/2.2	Viro B	31	25.9
		CSF0864(20dpi)/2.3	Viro C	25	21.83
		CSF1060(14dpi)/2.2	Viro D	21	16.15
		Porcine blood negative	Viro E	undet.	undet.
		CSF1060(14dpi)/2.2	Viro F	28	22.08
		CSF0864(20dpi)/2.3	Viro G	25	20.46
		CSF1060(14dpi)/2.2	Viro H	25	17.74

## Data Availability

The original contributions presented in this study are provided within the manuscript.
